# Neurosteroid Receptor Modulators for Treating Traumatic Brain Injury

**DOI:** 10.1007/s13311-023-01428-7

**Published:** 2023-08-31

**Authors:** Todd A. Verdoorn, Tom J. Parry, Graziano Pinna, Jonathan Lifshitz

**Affiliations:** 1 NeuroTrauma Sciences, LLC, 2655 Northwinds Parkway, Alpharetta, GA 30009 USA; 2 Psychiatric Institute, Department of Psychiatry, University of Illinois at Chicago College of Medicine, 1601 W. Taylor Street, Chicago, IL 60612 USA; 3 Department of Psychiatry, University of Arizona College of Medicine – Phoenix, 475 N. 5th Street, Phoenix, AZ 85004 USA

**Keywords:** Traumatic brain injury, Drug therapy, Neurosteroid, Progesterone, Allopregnanolone

## Abstract

Traumatic brain injury (TBI) triggers wide-ranging pathology that impacts multiple biochemical and physiological systems, both inside and outside the brain. Functional recovery in patients is impeded by early onset brain edema, acute and chronic inflammation, delayed cell death, and neurovascular disruption. Drug treatments that target these deficits are under active development, but it seems likely that fully effective therapy may require interruption of the multiplicity of TBI-induced pathological processes either by a cocktail of drug treatments or a single pleiotropic drug. The complex and highly interconnected biochemical network embodied by the neurosteroid system offers multiple options for the research and development of pleiotropic drug treatments that may provide benefit for those who have suffered a TBI. This narrative review examines the neurosteroids and their signaling systems and proposes directions for their utility in the next stage of TBI drug research and development.

## Introduction

TBI initiates a variety of pathological processes that often result in long-term damage and dysfunction. Even mild TBI can disrupt neurovascular function, and trigger necrosis, apoptosis, and inflammation within central nervous system (CNS). It is becoming increasingly clear that inflammation extends well beyond the brain to peripheral tissues and organs resulting in a detrimental disease state that persists long after the initial injury event (for review, see [1]). The complex and varied symptoms experienced after a TBI result from a cascade of tightly interacting biochemical pathophysiological mechanisms locked in enduring feedback loops. It is unlikely that highly selective, single-target drugs could effectively disrupt the whole spectrum of pathology, thereby, markedly limiting their potential benefit. Thus, the use of pleiotropic, multi-mechanism operating drugs may provide better therapeutic opportunities.

Neurosteroids (steroids produced within the brain), also known as neuroactive steroids when they reach and act in the brain after being produced in the peripheral glands, derive from cholesterol or steroid hormones through a series of biosynthesizing enzymes expressed in the nervous system of vertebrates (for reviews of the history of neurosteroids, see [2–4]). Neurosteroids play a fundamental role throughout development in addition to brain disease and injury [5–12]. In fact, neurosteroid production is upregulated in response to nervous system trauma [13–15], an effect thought to be an endogenous pathophysiological response to brain injury. The link between neurosteroids and TBI was found in an early study by [16], which showed that normal estrous cycling female rats exhibited less edema (brain water content) following controlled cortical impact brain injury than males and pseudopregnant females had the greatest degree of protection against brain injury-induced edema, suggesting that progesterone is neuroprotective following TBI. The role of progesterone for treatment of experimental TBI has been demonstrated in many preclinical studies (reviewed by [17–20]). Extending from the preclinical observations of the protective role of progesterone in TBI, other neurosteroids including allopregnanolone, estrogen, and testosterone have shown activity in models of brain injury [21–25].

It is important to note that multiple neuroactive steroids have been tested clinically in TBI (e.g., pregnenolone and allopregnanolone; see NCT00623506, NCT01336413, and NCT01673828, Clinicaltrials.gov and [26] and some have been approved for use in CNS indications. IV allopregnanolone, also known as brexanolone, is FDA-approved for use in post-partum depression patients, with benefit likely attributed to its positive allosteric modulatory actions on multiple subtypes of gamma-amino-butyric acid (GABA)-A receptors, including those containing delta-subunits (thought to be extrasynaptic; for reviews, see [27, 28]). Recent evidence suggests that allopregnanolone may also be useful for post-concussive symptoms and post-traumatic stress disorder (PTSD) suffered after injury as evidenced by its improvement of injury-related GABA_A_ receptor signaling abnormalities, the abnormal pro-inflammatory cytokine profile, the low levels of cerebrospinal fluid, and circulating allopregnanolone in patients and reducing activity in anxiety-related brain circuitry [29, 30]. Another recently approved positive allosteric modulator of GABA_A_ receptors, ganaxolone, is a neuroactive steroid synthetic analog approved for the treatment of cyclin-dependent kinase-like 5 deficiency disorder (for review, see [31]) that has been shown to improve neuroinflammatory endpoints in a commonly used model of multiple sclerosis [32] and behavior in stress-induced models of PTSD and depression [33]. Taken together, there is robust preclinical and some clinical evidence that treatment with neuroactive steroids can improve some brain pathologies. Because of their anti-inflammatory, neuroprotective, neurotropic, and behavioral effects, neuroactive steroids may have therapeutic utility in the treatment of TBI.

Given the wealth of neuroprotective evidence for progesterone in preclinical models of TBI, progesterone was evaluated in multiple Phase 2 studies in TBI patients (mostly moderate to severe injury), showing some positive signals [34, 35]. However, progesterone did not produce significant therapeutic benefit in two large Phase 3 trials in patients with moderate to severe TBI [36, 37]. Follow-up analyses of these trials reveal that failure could have resulted from trial design issues including progesterone dose and regimen selection, enrollment of a patient majority with severe and heterogenous injury, adequacy of endpoints, issues discussed in a later section of this review [38–41]. Therefore, it seems probable the hypothesis that progesterone treatment could improve outcomes in TBI patients was not properly tested in the above two Phase 3 studies.

With recent advances in both preclinical and clinical neurosteroid research and approval of products for CNS conditions, neuroactive steroid therapeutics continue to hold promise as TBI treatments. Of particular importance is the pleiotropic pharmacology of neuroactive steroids, positively impacting multiple pathophysiological processes known to be associated with TBI such as systemic and neuroinflammation, cerebrovascular inflammation, edema, apoptosis, axonal damage, oligodendrocyte loss, synaptic loss, and neurodegeneration. This review provides an overview of neuroactive steroid candidate therapies for TBI and their molecular mechanisms, and presents new directions for neuroactive steroid discovery and development.

## Neurosteroid Biochemistry and Pharmacology

The nervous system’s ability to produce neurosteroids de novo from cholesterol that is converted into the key precursor steroid, pregnenolone, arose from the discovery that the key regulatory enzyme, a cytochrome P450 variant encoded by the CYP11A1 gene, was expressed in the brain [42]. This cytochrome P450, known as CYP450scc, is responsible for cleaving cholesterol side chains normally expressed in steroidogenic glandular tissue (e.g., adrenal glands under the control of ACTH), within the mitochondria of peripheral nerves as well as central neurons and glia of the cerebellum and other brain regions [43, 44]. Importantly, it was found that pregnenolone-derived neurosteroids remained constant in the brain despite removal of sources of systemically derived steroids, demonstrating the brain as their source (reviewed by [45–47]). The expression of the key neurosteroid synthesizing enzymes at different ages and within various brain regions has been characterized [48, 49]. CYP450scc expression was found to be generally constant in the post-natal period and distributed throughout the brain.

**Fig. 1:**
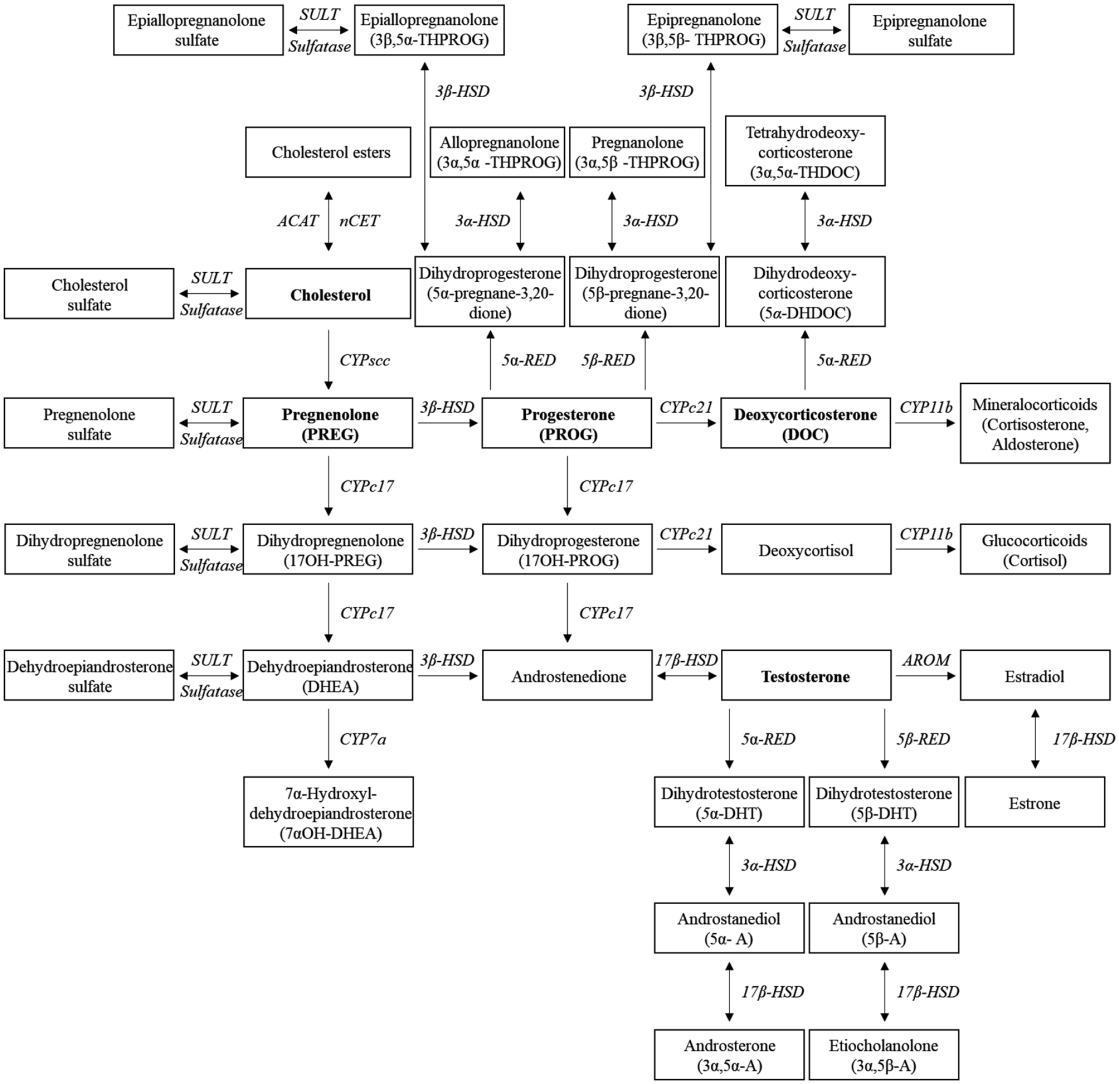
Biochemical network of neurosteroids and related molecules. Biosynthesis of neurosteroids. ACAT, acyl coenzyme A cholesterol acyltransferase; AROM, aromatase; CYP, cytochrome P450 hydroxylase; CYPscc, cytochrome P450 side-chain cleavage; HSD, hydroxysteroid oxidoreductase/dehydrogenase; nCEH, neutral cholesterol ester hydrolase; RED, reductase; SULT, sulfotransferase

Neurosteroids are generated through the action of multiple aromatase, hydrolase, and reductase enzymes in the cholesterol biosynthetic pathway (Fig. 1). Based on their chemical structures and precursors, neurosteroids are classified as follows:

(A) Pregnane neurosteroids, which are mainly progesterone derivatives, such as allopregnanolone, pregnanolone, and allotetrahydrodeoxycorticosterone
(B) Androstane neurosteroids, which are derived from testosterone, such as androstanediol, etiocholanone, and estrogens
(C) Sulfated neurosteroids, such as dehydroepiandrosterone sulfate and pregnenolone sulfate

The production of endogenous steroids is generally regulated by the hypothalamic-pituitary axis effects on steroidogenic tissues including gonads and adrenal cortex. Due to their lipophilicity, they can easily reach the brain via the circulation, penetrating the blood–brain barrier (BBB). However, certain steroid molecules, like pregnenolone sulfate, can be substrates for influx and efflux transporters to facilitate access to the brain [50].

One striking interpretation derived from the biosynthetic pathway of neurosteroids is the wide variety of endogenous molecules comprising this network and the complex biochemical control mechanisms associated with their production and degradation. Direct signaling roles for “prominent” components are based partly on interactions with specific receptors and their status as “action-at-a-distance” hormones. However, any of these may have critical roles in the pathophysiology of TBI.

Nuclear hormone receptors (NHR) [51, 52] are major molecular targets for a variety of metabolites (i.e., amino acid as well as fatty acid derivatives, porphyrins, and terpenoids) and other neurosteroids including the sterol family of molecules of which neurosteroids such as progesterone, allopregnanolone, pregnanolone, tetrahydrodeoxycorticosterone, and pregnenolone are members. Neurosteroid ligand-NHR interactions are linked to transcriptional control of gene expression and their activity can have long-lasting influence on cellular physiology and metabolism across a wide variety of tissues, including brain and cerebrovasculature. Target receptors for the array of neurosteroids include the group (or subfamily) 3 A-C such as estrogen receptors (NR3A1 and 2), estrogen-related receptors (NR3B1 and 2), glucocorticoid (NR3C1), mineralocorticoid (NR3C2), progesterone (NR3C3, receptors A and B from the same gene), and androgen (NR3C4) receptors [52]. Neurosteroids may also interact with other NHR subfamilies.

Neurosteroids also can interact with membrane bound receptors. For example, progesterone activates receptors in the membrane-associated progesterone receptor family (PGRMC1, PGRMC2, neudesin, and neuferricin), which are all expressed in the CNS and activate JAK/STAT and other intracellular signaling pathways (reviewed by [53, 54]). Short-term effects of neuroactive steroid treatments could also be mediated by their ability to directly modulate the activity of neuronal ion channels. Neurosteroids such as allopregnanolone (brexanolone) and ganaxolone interact with GABA_A_ receptors containing gamma subunits (synaptic GABA_A_ receptors) as well as delta subunits (extrasynaptic GABA_A_ receptors), acting as positive allosteric modulators (reviewed by [31, 55]). Positive allosteric modulation of the delta subunit-containing receptors increases tonic inhibition upon GABA binding. Some neurosteroids also act as N-methyl-D-aspartate (NMDA) receptor modulators (reviewed by [56]). Certain 3α-hydroxysteroids, such as RU5135, exhibit GABA_A_ receptor antagonist properties [57, 58]. Pregnanolone and pregnenolone sulfated neurosteroids modulate NMDA receptors, although in different ways [59–61]. While the action of pregnenolone sulfate is subunit dependent, pregnanolone sulfate mainly inhibits tonic-mediated NMDA receptor neurotransmission, which has been associated with neuroprotective effects [62, 63]. Together, this variety of short- and long-term actions offers mechanisms that could address neuronal pathology from multiple biochemical and physiological modalities.

## Progesterone and Progesterone Receptors in TBI

There is a large body of evidence on neuroprotective and pleiotropic actions of progesterone through its receptors (intracellular progesterone receptor and PGRMC1) in TBI (reviewed by [20, 64, 65]). Of interest is the dynamic nature of progesterone levels in the brain [66] as well as the expression of receptors in response to brain injury [14, 67, 68]. The critical role of the progesterone receptor in brain injury is highlighted in ischemic stroke where neuroprotection is reduced in neuron-specific progesterone receptor conditional knockouts [69]. Mechanistic effects of progesterone treatment on neuroprotection and reduction of inflammation after TBI have been reviewed [70–72]; however, the precise and relative role of the various progesterone receptor isoforms in neuroprotection has yet to be elucidated [71]. In addition to intracellular progesterone receptor-mediated effects on transcription, other pathways including the Nrf2/ARE, Src-Erk1/2 cascade, and Akt pathways as well as miRNAs may be involved in progesterone-mediated neuroprotection [64, 73–75], potentially affecting neural progenitor cell cycling [76]. It is important to note that levels of circulating progesterone following TBI may be quite high [38]. Thus, the use of neurosteroids that may produce optimal progesterone receptor stimulation under injury conditions (i.e., a partial progesterone receptor agonist) may be needed to avoid receptor over-activation.

Additional pharmacologic complexity is conferred by interactions with multiple additional receptors. Progesterone interacts with high affinity on multiple other NHR subtypes (e.g., different progesterone favoring NHRs and glucocorticoid receptors), and it appears to activate membrane receptors having different structural and functional features [53]. Similar receptor promiscuity exists for estrogen and testosterone. Some neurosteroids including allopregnanolone and pregnanolone have potent, direct modulatory activity on ion channel neurotransmitter receptors including GABA_A_ and NMDA receptors. This wide variety of biologically relevant interactions across the landscape of endogenous neurosteroid molecules suggests that there are likely additional neurosteroid receptor and ion channel interactions that have not been discovered. In any case, the neurosteroid class of molecules is broad-spectrum modulators of brain function that offer multiple options for development and testing of pleiotropic TBI treatments.

At least partly due to the multiplicity of receptor interactions, progesterone is considered an archetype of pleiotropic neurosteroid molecules. Multiple studies revealed transcriptional effects of classical nuclear progesterone receptors A and B, as well as putative membrane progesterone receptors including seven transmembrane domain (7TMPRβ) and membrane-associated 25-Dx receptors [65]. Progesterone and allopregnanolone were shown to bind to membrane progesterone receptors in mammalian cells acting as agonists, thus activating stimulatory G-protein-coupled receptors and decreasing apoptosis and necrosis by activation of mitogen-activated protein kinase/extracellular signal-regulated protein kinase (MAPK/ERK) and Akt pathways [77]. The direction of the progesterone receptor-mediated actions is routinely supportive of the neuroprotective effect of progesterone that involves reducing BBB dysfunction, amelioration of neuroinflammation, and improvement in myelination [65, 78, 79]

Widely-reproduced preclinical efficacy results in rat and mouse TBI models [18, 80–86] led to the evaluation of progesterone as a treatment for TBI in multiple Phase 2 and Phase 3 clinical trials [34–37]. Whereas two initial Phase 2 studies [34, 35] showed promising results with progesterone treatment, subsequent, larger Phase 3 studies [36, 37] failed to show benefit over placebo treatment. One interpretation of these results is that progesterone treatment is simply ineffective in human patients suffering a severe TBI, especially regarding death or vegetative state [87]. However, post-trial analyses [38, 39, 41] suggest there were multiple issues outside of progesterone’s efficacy that contributed to the negative outcome. Among the most notable reasons was the selection of severe TBI patients for the trial. These patients typically suffer multiple non-CNS injuries and are given a wide range of drug treatments to treat these injuries. This clearly could have impeded the ability to observe a treatment effect of progesterone. Furthermore, the choice of progesterone dose level and regimen was not based on careful tests of target engagement in humans, and therefore had a high probability of being inappropriate. Progesterone exhibits an inverted U-shaped dose–response relationship [88] in preclinical studies, and using the wrong dose level in the trial could certainly have resulted in the observed outcomes even though a proper dose of progesterone could have been effective. Based on these and other considerations thoroughly discussed by [38, 39, 41], the SYNAPSE and PROTECT trials likely did not fully test the hypothesis that progesterone treatment is beneficial for TBI patients.

## Estrogen and Estrogen Receptors in TBI

Sex-related responses to TBI have been the subject of both preclinical and clinical investigation [84, 89]. While estrogen production by peripheral organs is widely known, the role of estrogen as a neurosteroid is highlighted by its production by astrocytes [90]. Female mice subjected to TBI have improved survival and sensorimotor function compared to males and ovariectomized females [91]. Systemic administration of estrogens has also been shown to reduce neurological deficits in male mice following TBI [92], potentially through inhibiting activation of microglia and astrocytic inflammatory responses with reduced expression of inflammatory and pro-apoptotic genes [92].

It is well known that estrogen serves a neuroprotective role, likely due to reduced neuroinflammation as evidenced by estrogens reducing clinical scores in models of experimental autoimmune encephalomyelitis [93–95]. Estrogen-mediated neuroprotection also involves antiapoptotic activity through inhibition of excitotoxicity [96–98]. There is substantial evidence for estrogen-induced neuroprotection in a variety of neurodegenerative disease models including Alzheimer’s disease (see reviews by [99, 100]), Parkinson’s disease (see reviews by [101, 102]), and in stroke (see reviews by [103–106]). Of note, estrogen efficacy is lost in reproductively senescent female mice, potentially due to a loss of expression of both nuclear hormone estrogen receptor subtypes, ERα and ERβ. Estrogen-mediated neuroprotection has also been shown to involve activation of the membrane bound G protein-coupled estrogen receptor, GPR30 in experimental stroke [107]. Taken together, neuroactive steroids with estrogen receptor agonist activity at ERα, ERβ, and/or GPR30 may provide therapeutic benefit in the context of TBI. However, estrogen responsiveness may be reduced with age [108].

## Androgens and Androgen Receptors in TBI

The functional effects of modulating androgen receptors (AR) in TBI are poorly understood clinically, are less clearly associated with neuroprotection, and depend on the system under study [109]. It is well known that males have high risk for cerebrovascular disease/stroke [110], suggesting some interaction of androgens to neurovascular function. Whereas male rodents subjected to ischemic stroke or neonatal ischemia tend to have greater brain damage than age-matched females [111], a potential neuroprotective role of estrogen or other neurosteroids in neuroprotection cannot be ruled out. The direct role of androgens in the injured CNS has received limited attention. Pointing to the direct role of testosterone on neurotoxicity, Yang and coworkers found that supplementation of testosterone in castrated male rats led to increased lesion size following ischemic stroke compared to testosterone-depleted males [112]. Their work also showed that testosterone enhanced glutamate excitotoxicity in cultured HT22 cells. While this evidence points to an adverse role for androgen signaling in brain injury (i.e., stroke), clinical findings suggest that testosterone decline associated with aging might have an adverse effect on cognition and can be associated with depression and anxiety; similar mood-related observations have been confirmed in patients taking finasteride [113].

In TBI patients, Renner et al. [114] found no association between sex and outcomes while Styrke and coworkers [115] identified greater disability in women vs. men. In mice subjected to TBI, neuroinflammatory changes, astrogliosis, cell death, and lesion size were greater in males than females [116]. However, Chen and coworkers [117] found that AR knockout mice display increased astrogliosis and necrosis as well as relatively worse neurological deficits than their AR-competent counterparts. Adding to the confusion of whether AR signaling is involved in post-TBI recovery, the expression of ARs decreases with blast-injury in the hippocampus [118] and varies between male and female mice at 3 days post-TBI with males having reduced and females having increased AR expression post-injury [119]. These authors advocate for the relative expression of estrogen to AR levels as being the primary driver, switching to greater estrogen receptor signaling during recovery. While some studies suggest that withdrawal from androgen treatment reduces neurologic damage after insults caused by ischemic stroke, other studies indicate that the effects of testosterone on AR signaling is dose-dependent and can vary from promoting damage to neuroprotective effects [24, 119–122].

In addition to androgen-mediated effects on the neuropil, testosterone was shown to induce endothelium-dependent vasoconstriction of middle cerebral arteries [123], which could be, in part, mediated by ARs present in endothelial cells. Such vasoconstriction can exacerbate neurovascular reactions to injury. These mixed findings can be attributed to the fact that binding to cytosolic and/or membrane-bound ARs triggers a variety of signaling pathways, some of which might propagate neurotoxicity (e.g., intracellular Ca^+2^ influx, induction of pro-apoptotic genes and oxidative stress, and compromised mitochondrial function), while some are responsible for neuroprotection (e.g., activation of MAPK/ERK pathway, anti-inflammatory pathways) [24, 122, 124–126]. Thus, the relative role of androgen signaling in pathology and potential therapeutic benefits of androgen-related therapeutics (agonists or antagonists) after brain injury is unclear are indeed complex.

## Mineralocorticoids and Mineralocorticoid Receptors in TBI

The mineralocorticoid receptor (MR) is a NHR that is activated by both glucocorticoids and the mineralocorticoid, aldosterone. Under physiological conditions, brain glucocorticoids are in high abundance relative to aldosterone and act on MRs [127], often occupying the receptor under physiologic conditions where glucocorticoid concentrations greatly exceed circulating aldosterone levels [128, 129]. In addition to their adverse metabolic effect profile when used chronically, there is considerable evidence that glucocorticoid use in severe TBI patients offers no improvement [130]. Interestingly, GR activation can aggravate brain injury in a variety of conditions [131–133] and GR antagonism rescues neuronal loss in the hippocampus post-TBI [134]. The endogenous ligand, aldosterone, is only marginally brain penetrant due to P-glycoprotein efflux [135]; however, certain areas of weak BBB function allow for aldosterone to penetrate and exert actions related to blood pressure and blood volume control where 11-beta-dehydrogenase 2 (HSD2) is expressed to limit glucocorticoid levels at the MR, thereby improving aldosterone activity [136]. MR are distributed throughout the brain, with higher concentrations found in the limbic-frontocortical neurons, hippocampus, hypothalamus, cerebellum, and brain stem motor nuclei, with the nucleus solitarius likely most sensitive to circulating levels of aldosterone given their co-expression of HSD2 [136]. It is likely that MR activity in other brain regions outside of areas with a weak BBB is likely a function of glucocorticoid occupancy of the MR receptor and could contribute to neurotoxic effects of glucocorticoids, particularly under conditions of high stress [137].

The use of available MR antagonists has shed some light on the potential role of modulating MR activity in TBI. One obvious role of the MR in TBI pathology is the regulation of fluid balance and possible effects on edema that is often associated with injury. It is well known that MRs directly modulate the expression of the Na–K ATPase and subsequently the epithelial sodium channel to control hydrostatic balance in kidney and other tissues [138]. Whereas blockade of MRs is expected to reduce fluid retention systemically, MR antagonism does not appear to be effective in managing cerebral edema per se. There is evidence that MR antagonism may be helpful to reduce inflammation in endothelial cells [139]. Numerous studies indicate that modulating cerebrovascular MR activity could be beneficial in managing neurovascular function following brain injury [140–147]. Given the effects of MR antagonism on neurovascular inflammation, combined MR antagonist use with progesterone receptor (PR) agonism could be additive or synergistic at reducing cerebral edema and normalizing neurovascular function in TBI.

MR blockade by synthetic steroid MR antagonists may be anti-inflammatory within the nervous system. In addition to effects on edema, MR antagonism in the brain following TBI has been shown to have anti-inflammatory effects. MR stimulation is pro-inflammatory in non-brain tissues [148, 149]. NF-κB translocation and pro-inflammatory cytokine production was increased by aldosterone in BV-2 microglial cells, effects blocked by spironolactone [150]. In stroke-prone spontaneously hypertensive rats supplemented with NaCl/stroke prone diet for 19 weeks, MR antagonism with eplerenone improved survival and cerebral injury in ischemic and hemorrhagic strokes [151]. In a model of postoperative cognitive impairment, MR expression was activated in the hippocampus CA1 along with expression of inflammatory cytokines, all paralleling cognitive impairment. In this study, MR antagonism improved cognitive dysfunction and reduced hippocampal MR overexpression as well as inflammatory cytokine levels. In a model of dorsal root ganglia inflammation, eplerenone potentiated glucocorticoid anti-inflammatory activity, reduced glial fibrillary acidic protein (GFAP) levels, and improved behavioral nociceptive responses in rats [152]. While MR agonism plays a role in normal brain physiology, the above evidence suggests that synthetic steroids possessing MR antagonist activity could be therapeutically beneficial in TBI.

## Role of Liver X Receptor and Retinoid X Receptor in TBI

The potential roles of other NHRs in TBI are still being elucidated. The liver X receptor (LXR) has a role in brain function where its activation modulates inflammation and lipid metabolism. Genetically modified mice lacking one subtype of LXR show a variety of brain-related pathologies, indicating it plays a critical role in brain cell health and survival [153]. A sterol-based agonist of LXR has shown beneficial effects in a mouse model of TBI [154]. A different LXR agonist also improved learning and memory and prevented axonal degeneration in mice following repetitive mild TBI [155]. Genetically modified mice lacking LXR did not improve with LXR agonist treatment, confirming the role of LXR in the effect of drug treatment.

TBI has been associated with disruption of lipid metabolizing enzymes [156] resulting in altered levels of cholesterol end-products and inflammatory mediators [157]. Therefore, LXR activation may provide both anti-inflammatory action and stabilization of lipid metabolism. Since cholesterol represents the upstream precursor of neuroactive steroids, including progesterone, the effects of LXR agonists on neurosteroidogenesis may be worth further investigation.

Additional new treatments for TBI may arise from modulation of the retinoid X receptor (RXR) retinoid-responsive system. RXR is activated by retinoic acid but is distinct from the classic retinoic acid/vitamin A receptor, RAR. Bexarotene is a 3rd generation retinoid compound that is a selective agonist of RXR without effect on RAR. It is in clinical use as an anti-tumor agent. A series of studies have reported bexarotene treatments facilitated axon sprouting and cognitive performance in mouse TBI models that have been mechanistically ascribed to facilitation of BDNF signaling [158], inhibition of apoptosis [159], and via modulation of PPAR$\gamma$ signaling [160]. Due to RXR’s wide-ranging control of a variety of inflammatory and cell survival pathway, it seems likely that each of these mechanisms contributes to the efficacy of bexarotene and potentially other RXR agonists that are under active development.

## Therapeutic Exposure to Neuroactive Steroid-Based Drugs

An often overlooked consideration in research on neuroactive steroid treatments for TBI is the concentration of endogenous neurosteroids at relevant receptors and their relative affinities, and its relation to efficacy. Typical plasma and brain concentrations of most types of endogenous neurosteroids are well below 1 ng/mL [69]. Although we expect neurosteroid concentrations to fluctuate in the receptor local environment as signaling waxes and wanes, endogenous concentrations are 100–1000-fold lower than the peak concentrations of progesterone (for example) expected to occur after an 8 mg/kg level dose of progesterone often used in neuroprotection studies. Variations in circulating and brain progesterone and other hormones after TBI should also be considered when developing neuroactive steroid therapeutics [66, 161]. Furthermore, MRs are modulated by glucocorticoids whose physiologic concentrations are much greater than aldosterone in most brain regions [128]. In physiological conditions, the neurosteroid system is clearly tuned for responding to subtle concentration change. Indeed, progesterone-based oral contraceptives involve changes of less than 1 nM in plasma concentrations. We can only conclude that the changes in brain and plasma concentrations accompanying neuroprotective dose levels would swamp an otherwise tightly controlled biochemical system.

Mechanistic investigations of neuroactive steroid actions are often interpreted as direct effects on NHRs or related neurosteroid receptors, but it is much more likely that biochemical outcomes are the result of widespread disruption of a finely tuned homeostatic system. While this view generally supports the conclusion of neuroactive steroid pleiotropism after therapeutic dose levels, it also means that our current understanding of mechanisms could be quite inaccurate and incomplete, including effects on endocrine and peripheral inflammatory mechanisms induced by TBI that help perpetuate neuroinflammation chronically [162–166]. It is important to evaluate not only whether acute neuroactive steroid use could improve long-term patient outcomes, but also to examine whether patients with chronic post-TBI deficits could benefit with chronic neuroactive steroid therapy.

## New Directions

The pharmacology of neuroactive steroids and the wide variety of mechanisms modulated by them provide a wealth of opportunity to generate new, improved treatment strategies for both acute and chronic TBI. Potential breakthroughs could arise from repurposing of available neuroactive steroid molecules to discovery and development of structural analogs having novel physicochemical and pharmacological properties (i.e., enhanced solubility, relative impact on various NHRs, enhanced potency, reduced off-target effects). Based on the foundation of preclinical efficacy, partially promising clinical results and the ability to address known translational pitfalls, improving on progesterone represents a well-founded strategy. For example, the collaboration between the Stein laboratory and the Emory Institute of Drug Discovery has developed multiple water-soluble analogs of progesterone that could address drug dosing and delivery issues associated with the use of progesterone itself [85, 167]. Analogs with different relative affinities for various NHR types or others that favor activation of different signaling pathways may generate better highly pleiotropic neuroprotective or neuro-restorative actions. Finally, the potential for an inverted U-shaped dose–response [38] represents a drawback of progesterone that may be addressed by progesterone analogs that have partial agonist actions and interact with other NHRs that impact TBI pathologies.

Due to the highly interconnected biochemical network that generates multiple active neurosteroids, many having some neuroprotective or neurorestorative effects, drug interventions that change the activity of metabolizing enzymes or otherwise alter the balance of enzyme substrates may offer a different approach for exploiting neuroactive steroids for TBI treatment purposes. For example, it seems likely that inhibition of the 5-$\alpha$-reductase that metabolizes progesterone to dihydroprogesterone results in a build-up of progesterone that itself could provide neuroprotection. Considering the concentration issues described above, the pharmacologic levels of progesterone resulting from an externally applied dose could also increase the levels of neurosteroids downstream from progesterone in the biochemical pathway including the GABA_A_ receptor modulator, allopregnanolone. This widespread change in neurosteroid levels would not only alter the activity of metabolizing enzymes but may also trigger compensatory changes in the levels of receptors, metabolizing enzymes, and downstream effectors. Based on our current, limited understanding of this complex system, it is possible that pleiotropic neuroactive steroids that affect multiple NHRs may lead to improved therapeutics for TBI.

## Conclusions

As drug discovery and development science embraces pleiotropic approaches for achieving neuroprotection and neurorestoration, pharmacologic modulation of the neurosteroid system represents a fertile area for continued research. Neuroactive steroids are intimately involved in regulation of inflammation, cell health, fluid balance, and growth factors, all mechanisms known to be negatively impacted by TBI. Moreover, the complex interplay of neuroactive steroid synthesis and receptor modulation suggests that we still only have a surface-level understanding of optimal ways to modulate this system for maximum therapeutic benefit.
